# Solitary Calvarial Metastasis As the First Presentation of Urothelial Bladder Cancer

**DOI:** 10.7759/cureus.17257

**Published:** 2021-08-17

**Authors:** Simona Bistazzoni, Sara Bonetto, Silvio Domenico Bellocchi

**Affiliations:** 1 Neurosurgery, Sant'Anna Hospital, Como, ITA; 2 Pathology, Sant'Anna Hospital, Como, ITA

**Keywords:** calvarian metastasis, bladder carcinoma, skull, skull bladder metastasis, skull metastasis surgery

## Abstract

The most common sites of metastasis from urothelial carcinoma of the bladder include the lymph nodes, bones, lungs, liver, and peritoneum. Bladder carcinoma metastases of the head are uncommon. In the English literature, only a few cases of bladder carcinoma metastasis to the skull base have been reported, and no cases of metastasis to the calvaria have been reported previously.

We present the first case of calvarial metastasis from bladder carcinoma that preceded the diagnosis of the primary tumor.

Bladder calvarial metastasis as the first evidence of a primary tumor is a rare event and a sign of an advanced stage of the disease. Therefore, adequate staging is essential to ensure appropriate treatment. Surgical treatment of calvarial injuries is usually secure.

## Introduction

Skull metastases are rare and difficult to diagnose due to their asymptomaticity; therefore, there are more published autopsy series than clinical case reports. Occasionally, a solitary skull metastasis may be the only evidence of metastatic disease [1]. Skull metastases manifest in 15%-25% of all systemic cancer [2] and can occur via hematogenous spread, direct extension from an intracranial primary tumor, leptomeningeal extension, and leukemic or lymphomatous infiltration. Metastases that occur via hematogenous spread may be from various malignancies located all over the body, including the breast (55%), lung (14%), prostate (6%), lymph node (5%), and others (20%) [3]. Bladder carcinoma metastases of the head are uncommon, and their most frequent sites are the brain, supraclavicular nodes, neck nodes, and the skull [4].

We present the first case of calvarial metastasis from bladder carcinoma that preceded the diagnosis of the primary tumor.

## Case presentation

**Figure 1:**
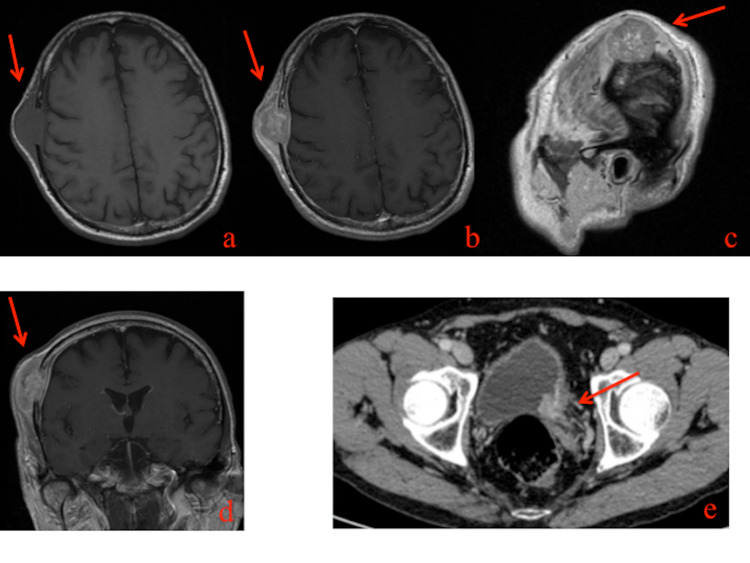
Preoperative MRI and abdomen CT scan Pre-contrast T1 axial (a), post-contrast T1 axial (b), post-contrast T1 sagittal (c), and post-contrast T1 coronal (d) MRI sequences documented an expansive lesion (28x20x38mm diameter) of the right frontotemporal cranial theca. The lesion caused complete lysis to the thickness of the cranial bone. Post-contrast abdomen CT scan showed the presence of a left postero-lateral bladder wall lesion (e).

**Figure 2:**
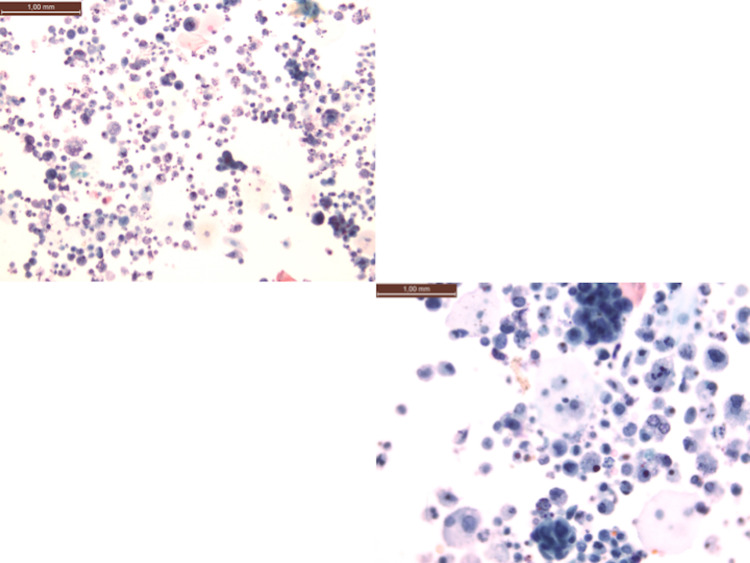
Urinary cytology Urinary sediment with isolated elements and morular aggregates of carcinoma cells mixed with inflammatory elements (Papanicolaou, 400x)

A 65-year-old man came to our attention in October 2018 due to the appearance of a right frontotemporal swelling with progressive growth for about a month and a half. In anamnesis, he had a single kidney. The patient’s neurological exam was negative. Contrast-enhanced brain magnetic resonance imaging (MRI) revealed a large lesion (28 x 20 x 38 mm) in the right frontotemporal cranial theca. The lesion had caused complete bone lysis involving the full thickness of cranial planking and extending widely into the subcutaneous soft tissues while extending approximately 4 mm into the extradural space. In the T1 sequence, the lesion was homogeneously intense and, after administration of contrast, it was characterized by inhomogeneous enhancement (Figure 1). As metastasis was suspected, the patient preoperatively underwent a total body computed tomography (CT) scan, which showed the presence of diffuse thickening of the left postero-lateral bladder wall and a solid endoluminal protrusion at the ureteral outlet, characterized by irregular endoluminal margins and discrete enhancement (Figure 1). Urinary cytology showed the presence of carcinoma cells (Figure 2).

The patient gave informed consent for the surgery and to the use of the data for scientific purposes. During the operation, which took place on July 11, 2018, the patient underwent gross total removal (GTR) of the head lesion and subtotal resection (SR) of the bladder lesion. The cranial lesion was of a hard-elastic consistency and was resected en bloc, involving bone margins apparently without disease. The dura mater was infiltrated so it was removed keeping very wide edges. Dural plastic surgery using NeoDura and cranioplasty with titanium mesh were performed. The temporal fascia and epicranial tissue over the lesion were removed.

During the transurethral cystoscopy, in the left postero-lateral bladder wall, a solid lesion of about 2 cm with a large implant base with significant periwound edema was identified. The lesion was transurethrally resected. The resection did not appear to reach healthy tissue.

**Figure 3:**
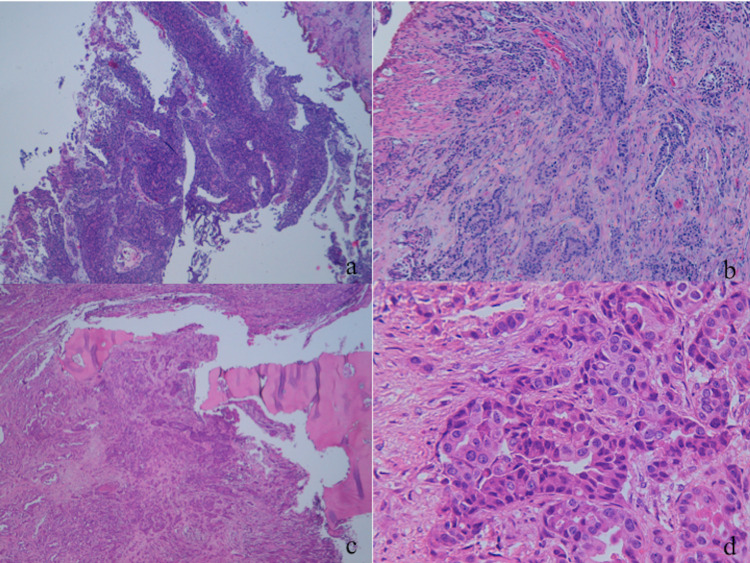
Bladder and cranial histological exam (a) High-grade urothelial carcinoma. (b) Detail at a greater magnification of the bladder lesion. (c) A cranial lesion with bone infiltration. (d) Detail at a greater magnification of the cranial lesion.

A histological exam of the cranial lesion revealed metastasis of poorly differentiated carcinoma with an immunophenotype compatible with urothelial origin (cytokeratin 7+, cytokeratin 20+, gata 3 -/+) and focal infiltration of the dura mater (Figure 3).

A histological exam of the bladder lesion revealed a high-grade urothelial carcinoma (gata 3+, chromogranin-, synaptophysin-) infiltrating the subepithelial connective tissue (t2 hg9). The results of molecular screening (fibroblast growth factor receptors (FGFR) mutation/translocation research) were negative for molecular alterations of FGFR3 (Figure 3).

The patient was discharged in good clinical and neurological conditions six days after surgery.

Oncoradiological screening (scintigraphic bone scan [December 5, 2018], chest-abdomen CT scan [January 25, 2019], and positron emission tomography [PET] scan [February 11, 2019]) confirmed the presence of a bladder wall lesion and revealed the presence of lesions at the level of the left hemi-mandible, right ethmoid, the middle third of the left femur, as well as 2-mm lung focalities, and lymphadenopathy at the level of the left common iliac artery and retroaortic artery.

Therefore, the patient underwent chemotherapy (carbo therapy and gemcitabine for four cycles) and radiotherapy to the left hemi-mandible (up to 30Gy in 10 fractions with the 3Dconf technique and 6MV photons) between March 3, 2019, and March 29, 2019, and to the middle third of the left femur (up to 20Gy in five fractions with the 3Dconf technique and 18MV photons) between March 18, 2019, and March 22, 2019, with an initial good response.

Radiological examinations showed good tumor control. PET scans (performed on June 4, 2019, and October 30, 2019) showed a significant lowering of the pathological accumulation of the radio drug, and the chest-abdomen CT scan indicated a reduction in the size of the known lesions.

**Figure 4:**
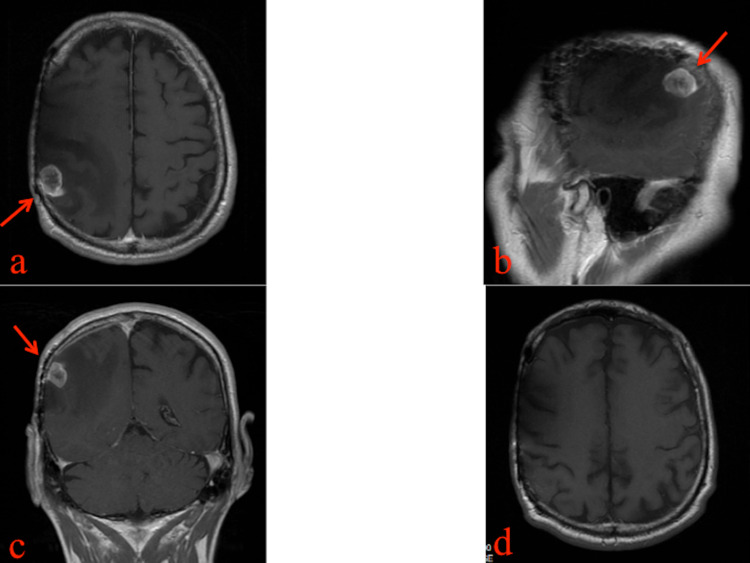
Pre and postoperative brain MRI Post-contrast axial T1 (a), post-contrast sagittal T1 (b), post-contrast coronal T1 MRI sequences (December 18, 2019) showed a metastatic, cortico-subcortical, right temporoparietal lesion with profuse edema. Postoperative post-contrast axial T1 MRI sequence (d).

In December 2019, the patient reported motor impairment in his left hand. MRI scan revealed a metastatic cortico-subcortical right temporoparietal lesion (Figure 4). On December 27, 2019, the patient underwent surgery to remove the brain lesion.

**Figure 5:**
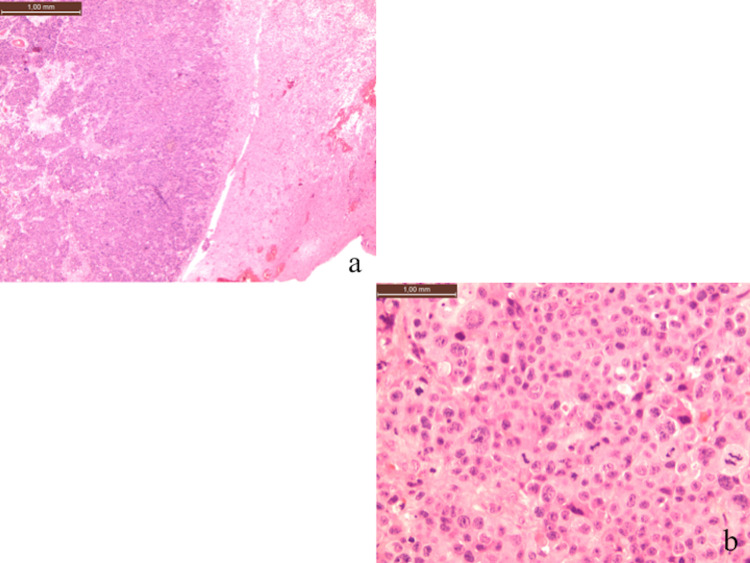
Histological examination of brain metastasis (a) Brain parenchyma with poorly differentiated epithelial metastases (hematoxylin-eosin, 100x). (b) Detail at a greater magnification of the metastases: epithelial elements with cytological atypia (hematoxylin-eosin, 400x). This population was found to be diffusely positive for cytokeratin 20 and cytokeratin 7 and focally positive for GATA3, consistent with the immunophenotypic profile of transitional cell carcinoma.

A histological exam confirmed the known diagnosis (Figure 5). The patient immediately showed an immediate improvement of the neurological deficit and was discharged three days after surgery in good clinical condition. The patient died two months later after suffering a heart attack.

## Discussion

The most common sites of metastasis from urothelial carcinoma of the bladder include the lymph nodes, bones, lungs, liver, and peritoneum. The skeleton is a common site of metastases for carcinoma of the bladder, with up to 27% of muscle-invasive transitional cell carcinomas presenting with bone metastasis [5]. As with any bones in the body, the skull can be a site for tumor metastasis. Cancer of the prostate, breast, and lung are the main source of skull metastases, accounting for more than 70% of secondary tumors of the skull [4]. Calvarial lesions may occur in the frontal, parietal, temporal, or occipital bones, in contrast to skull base lesions, which are localized in the orbital plates of the frontal bone, the greater or lesser wings of the sphenoid bone, the basisphenoid bone, the temporal bone (petrous portion), or the basioccipital bone. The calvaria consists of an inner table, bone marrow space, and an outer table. In particular, metastases to the calvarial bones usually involve all three skull layers [2].

In the English-language literature, only a few cases of bladder carcinoma metastasis to the skull base have been reported, and no cases have been reported of metastasis to the calvaria. Ogunyemi et al. reported the case of a 62-year-old patient with metastases of transitional cell carcinoma of the bladder to the sphenoid and cavernous sinus [4]. Nasi et al. presented the case of a 70-year-old man with metastases of urothelial carcinoma to the clivus and craniocervical junction [6]. Saldanha et al. reported the case of an 80-year-old man with metastases of anaplastic carcinoma of the bladder to the left petrous temporal bone [7]. Yildirim-Baylan et al. presented the case of a 52-year-old man with metastases of transitional cell carcinoma of the bladder to the left and right petrous temporal bones [8].

Bladder cancer typically spreads to the bone hematogenously. Hematogenous spread of bladder cancer can result from tumor infiltration into the vesical or prostatic venous plexuses and can bypass the major caval system to cause distant metastasis through the vertebral venous system [6].

Calvarial metastases are of less clinical importance than intraparenchymal ones, as most of the former are asymptomatic. When they grow, they can cause local swelling that is usually painless, but if they involve the outer table, periosteum, or dura mater, they may cause localized pain or may lead to compression of the brain, dural sinuses, and cranial nerves with meningeal irritation, neurological deficits, seizures, and increased intracranial pressure [1].

Patients presenting with calvarial metastases are often in an advanced stage of disease, and the diagnosis is often difficult due to their paucisyntomaticity; indeed, these metastases are frequently found in autopsies, and only a few studies have focused on their clinical presentation. The detection of calvarial metastases is also challenging in patients without known malignancy. Occasionally, a solitary skull metastasis may be the only evidence of metastatic disease. In our case, the patient had no urinary symptoms or any clinical manifestation to indicate the primary origin of the tumor [1].

Radiographically, skull metastases can be divided into lytic, sclerotic, and mixed types. Bladder carcinoma bone metastases are of the lytic type, as in our case. In general, purely osteolytic lesions are detected by plain radiography, but they may not be apparent until 30% demineralization occurs [9].

In clinical practice, a CT scan is commonly used to evaluate the type and the extent of bony abnormality while MRI provides additional information, such as the early involvement of the bone marrow, multiplicity, and the anatomical relationship to the brain and other intracranial structures; both modalities provide detailed morphology [4].

Radionuclide bone scan and [18F]-fluorodeoxyglucose positron emission tomography-computed tomography (18F-FDG PET-CT) are most commonly used to detect bony metastases at present. These exams reflect the osteoblastic reaction of bone to the presence of tumor cells. Although 18F-fluoride uptake, like that of methylene diphosphonate conjugated with 99m technetium (99mTc-MDP), depends on regional blood flow and osteoblastic activity, the better spatial resolution of PET and the favorable pharmacokinetic characteristics of 18F-fluoride render this tracer more sensitive for the detection of both lytic and blastic lesions. 18F-fluoride PET/CT has higher sensitivity, specificity, and accuracy in detecting bone metastases in urinary bladder carcinoma than conventional radionuclide bone scan [9].

Recently, Ryu et al. proposed that the routine use of arterial spin labeling (ASL) may help detect lesions in blind spots such as skull metastases. ASL is a non-invasive MRI technique used to measure cerebral blood flow (CBF) by inducing the magnetization of inflowing blood. ASL has an inherent advantage over other modalities because of its non-invasive and repeatable sequences without gadolinium. Therefore, it can be readily performed in patients with renal insufficiency or a history of contrast-mediated side effects [10].

Surgery is a safe palliative procedure with low morbidity and low mortality; therefore, it is recommended especially for accessible lesions that cause massive destruction of bone, dural infiltration, and neurological deficit, for painful masses, for solitary metastases, or to confirm/make a diagnosis [1].

## Conclusions

Bladder calvarial metastasis as the first evidence of a primary tumor is a rare event, and normally, the presence of a skull lesion is a sign of the advanced stage of the disease. CT and MRI allow adequate planning of surgical treatment. 18F-FDG PET-CT is the most accurate test for the diagnosis of bone metastases from urinary carcinoma. Adequate staging is essential to ensure appropriate treatment. Surgical treatment of calvarial injuries is usually secure.

## References

[1] Stark AM, Eichmann T, Mehdorn HM (2003). Skull metastases: clinical features, differential diagnosis, and review of the literature. Surg Neurol.

[2] Kotecha R, Angelov L, Barnett GH (2014). Calvarial and skull base metastases: expanding the clinical utility of Gamma Knife surgery. J Neurosurg.

[3] Mitsuya K, Nakasu Y, Horiguchi S (2011). Metastatic skull tumors: MRI features and a new conventional classification. J Neurooncol.

[4] Ogunyemi O, Rojas A, Hematpour K, Rogers D, Head C, Bennett C (2010). Metastasis of genitourinary tumors to the head and neck region. Eur Arch Otorhinolaryngol.

[5] Babaian RJ, Johnson DE, Llamas L, Ayala AG (1980). Metastases from transitional cell carcinoma of urinary bladder. Urology.

[6] Nasi D, Dobran M, di Somma L, Santinelli A, Iacoangeli M (2019). Sixth cranial nerve palsy and craniocervical junction instability due to metastatic urothelial bladder carcinoma. Case Rep Neurol.

[7] Saldanha CB, Bennett JD, Evans JN, Pambakian H (1989). Metastasis to the temporal bone, secondary to carcinoma of the bladder. J Laryngol Otol.

[8] Yildirim-Baylan M, Erdogmus N, Cureoglu S, Paparella MM (2011). Bladder cancer metastases to the temporal bone. Otol Neurotol.

[9] Chakraborty D, Bhattacharya A, Mete UK, Mittal BR (2013). Comparison of 18F fluoride PET/CT and 99mTc-MDP bone scan in the detection of skeletal metastases in urinary bladder carcinoma. Clin Nucl Med.

[10] Ryu KH, Baek HJ, Cho SB, Moon JI, Choi BH, Park SE, An HJ (2017). Skull metastases detecting on arterial spin labeling perfusion. Three case reports and review of literature. Medicine (Baltimore).

